# Tunable Kondo Behavior in a Bilayer Graphene Quantum Channel

**DOI:** 10.1021/acs.nanolett.6c01707

**Published:** 2026-09-16

**Authors:** Josep Ingla-Aynés, Serhii Volosheniuk, Talieh S. Ghiasi, Angelika Knothe, Kenji Watanabe, Takashi Taniguchi, Vladimir I. Fal’ko, Herre S. J. van der Zant

**Affiliations:** † Kavli Institute of Nanoscience, 2860 Delft University of Technology, Lorentzweg 1, 2628 CJ Delft, The Netherlands; ‡ Institut für Theoretische Physik, 9147 Universität Regensburg, D-93040 Regensburg, Germany; § Research Center for Functional Materials, 52747 National Institute for Materials Science, 1-1 Namiki, Tsukuba 305-0044, Japan; ∥ International Center for Materials Nanoarchitectonics, National Institute for Materials Science, 1-1 Namiki, Tsukuba 305-0044, Japan; ⊥ National Graphene Institute, 5292 University of Manchester, Booth Street East, Manchester M13 9PL, United Kingdom; # Department of Physics and Astronomy, University of Manchester, Oxford Road, Manchester M13 9PL, United Kingdom

**Keywords:** bilayer graphene, quantum point contact, Kondo effect, 0.7 anomaly

## Abstract

The interaction between itinerant electrons and localized spins is key to a wide range of electronic phenomena. We present conductance measurements of a quantum point contact (QPC) in bilayer graphene (BLG). Beyond the expected quantized conductance plateaus reflecting spin-and-valley degeneracy, we observe an additional plateau resembling the “0.7 anomaly”, exhibiting signatures of Kondo physics, with a Kondo temperature ranging from approximately 0.5 up to well above 5 K at zero magnetic field, corresponding to energies between 43 and 430 μeV. Because the QPC is spin-and-valley degenerate within our experimental resolution (110 μeV), according to the prevailing Kondo model, these results are consistent with a transition between 4-fold degenerate SU(4) and 2-fold degenerate spin-valley locked SU(2) interactions. Furthermore, we break the degeneracy of the lowest subband by an out-of-plane magnetic field and show that Kondo signatures remain, demonstrating the versatility of BLG QPCs.

## Graphical abstract

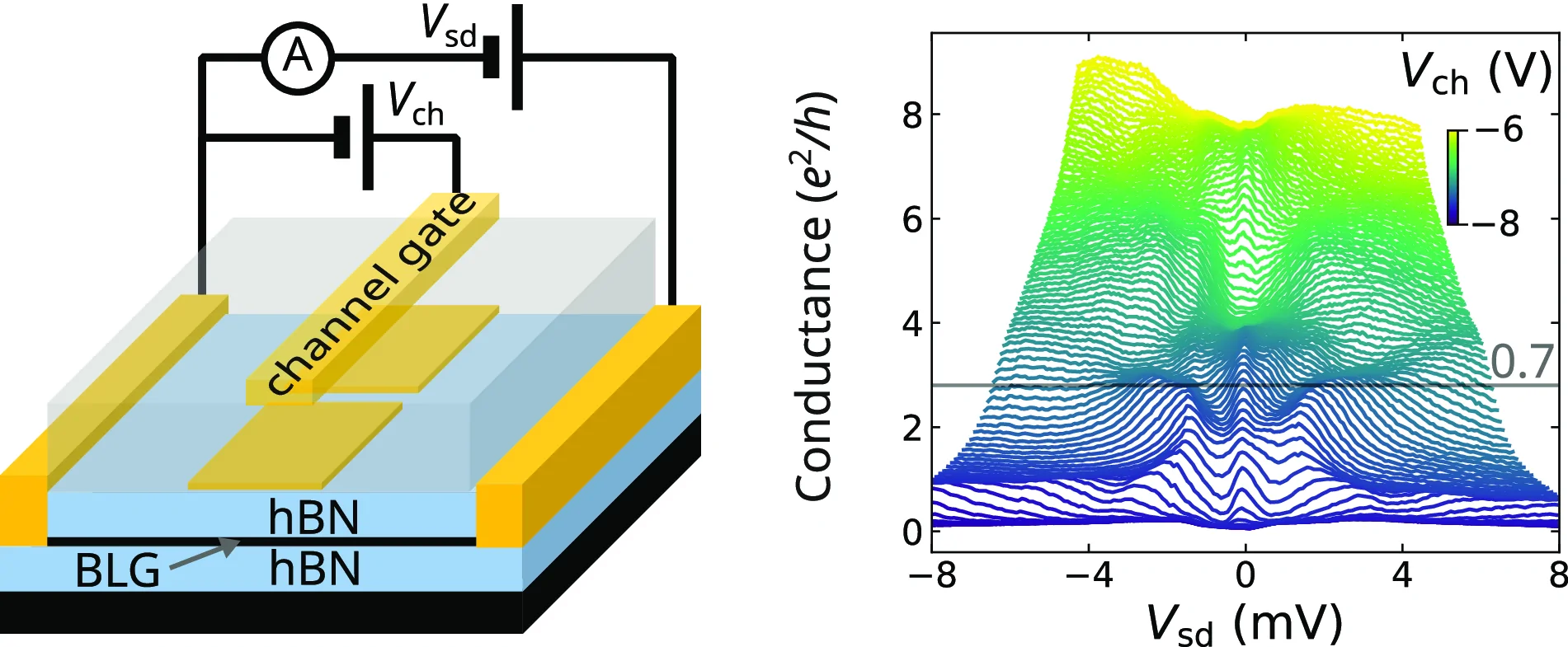

The Kondo effect describes the interaction between moving electrons and localized spins^1^ and is crucial for understanding physical phenomena such as superconductivity in heavy-Fermion materials.^2,3^ Its emergence in mesoscopic systems such as quantum dots^4–12^ and quantum point contacts (QPCs)^13–32^ has provided unprecedented control over these many-body states.^33^ In carbon nanotube quantum dots, spin and orbital degeneracy enhance Kondo interactions,^9,34^ and new features arise due to the emerging SU(4) symmetry.^35–40^

A QPC is a ballistic one-dimensional channel with a width comparable to the Fermi wavelength. As a result, its conductance (*G*) varies in steps of *k* × ν*e* ^2^/*h*, where *k* = 1, 2, ... is the subband number, ν = 2, 4 is the subband degeneracy, *e* is the electron charge, and *h* is the Plank constant.^41–44^ Furthermore, QPCs also show an additional plateau that, in two-dimensional electron gases without valley degeneracy (ν = 2), appears at *G* ≈ 0.7 × 2 *e* ^2^/*h*.^45–49^ Despite controversies,^49^ this level, called the “0.7 anomaly”, is associated with electron–electron interactions in the QPC, manifesting in Kondo- and Wigner-like physics, and continues to trigger extensive theoretical and experimental work.^13–32,50,51^

Bilayer graphene (BLG) offers a promising platform for the exploration of Kondo physics. As a tunable band-gap semiconductor,^52–56^ it has recently been introduced as a model system for the realization of electrostatically defined QPCs^57–64^ transmitting valley-polarized electron jets^65–71^ and hosting quantum dots^72,73^ showing Kondo physics.^11,12^ In addition, the emergence of correlated physics in BLG^74–76^ further motivates the exploration of Kondo phenomena in BLG-based devices.

The small spin–orbit (SO) gap of 40–80 μeV^77,78^ suggests that BLG QPCs may serve as an ideal platform for exploring spin-valley degenerate SU(4) Kondo physics.^10^ This regime has not yet been realized in BLG quantum dots, primarily because the Kondo temperature (*T*_K_) at zero magnetic field is approximately 1 K, corresponding to a Kondo energy *E*_K_ = *k*_B_ *T*_K_ ≈ 86 μeV, where *k*_B_ is the Boltzmann constant;^11^ a mesoscopic system with a higher *T*_K_ is required to explore SU(4) Kondo physics in BLG. Recent experimental work^31^ has revealed a subband in BLG QPCs consistent with the “0.7 anomaly”, opening new avenues for investigating Kondo physics in BLG to reach higher *T*_K_.

Here, we perform electronic transport experiments on a gate-defined QPC in BLG, showing spin-and-valley degeneracy (ν = 4), implying that any spin or valley splittings are lower than the experimental resolution of 110 μeV. The bias and carrier density dependence of *G* shows the activation of a level below the first subband with *G* ≈ 0.7 × 4*e* ^2^/*h* at finite bias, similar to the “0.7 and 0.85 anomalies” in GaAs-based QPCs,^47,49^ and a zero-bias peak (ZBP) that splits under the application of an in-plane magnetic field. We show that these observations are compatible with 2-fold SU(2) and 4-fold degenerate SU(4) universal Kondo scaling, with SU(2) activation temperatures ranging from approximately 0.5 to above 5 K when the carrier density in the QPC is changed. As a result, the SU(2) *E*_K_ becomes at least 4 times larger than any spin or valley splitting, indicating that conventional SU(2) Kondo scaling alone cannot explain our data and a spin-and-valley degenerate Kondo is expected. Thus, we argue that the symmetry of the Kondo effect may be changing from SU(2) to SU(4).^10,79^ We further observe that a moderate out-of-plane magnetic field (*B*_*z*_) breaks the subband degeneracy. Because the temperature and bias dependence at *B*_*z*_ = 0.6 T are consistent with Kondo physics, this result suggests that a Kondo regime with lower degeneracy may have been achieved with comparable Kondo temperatures (Section S6). The latter indicates that the thermal evolution of the lowest QPC level is robust to changes in the degeneracy, an unexpected finding because a drop in *T*_K_ is expected when the SU(4) Kondo state degeneracy is lifted.^9,34^ This result indicates that new theoretical work is required to understand the “0.7 anomaly” and its associated features in BLG QPCs.

The QPC geometry is sketched in Figure 1a. The device is a double-gated, hexagonal boron nitride (hBN)-encapsulated BLG heterostructure on a few-layer graphene back-gate^59,66^ where the upper and lower hBN flakes are 28 and 23 nm thick, respectively. The split gates, with a gap of approximately 50 by 600 nm (Section S2), were deposited on the upper hBN. The 300-nm-wide channel gate was deposited on a 60-nm-thick Al_2_O_3_ layer to tune the electrochemical potential of the QPC.

**Figure 1:**
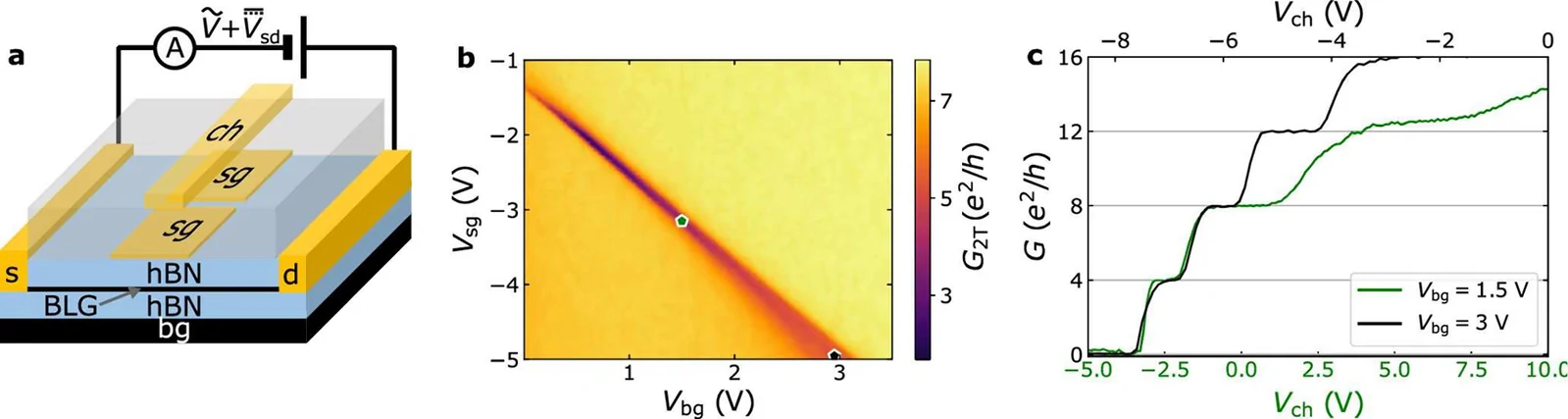
Gate-defined QPC in BLG. (a) Device geometry: The BLG is encapsulated between atomically flat hBN insulating layers, and the graphite back-gate (bg) is used in combination with two Ti/Au split gates (sg) to form the QPC. The channel gate (ch) is placed on an Al_2_O_3_ insulating layer to tune the QPC electrochemical potential. The contacts to the BLG are labeled as source (s) and drain (d). The circuit corresponds to the measurement geometry where *Ṽ* and *V*_sd_ are the applied ac and dc voltages, respectively. The ammeter (A) measures the source–drain current. (b) Two-terminal conductance (*G*_2T_) as a function of the bg (*V*_bg_) and sg (*V*_sg_) voltages. The QPC conductance *G* is obtained from *G*_2T_ by subtracting the contact and ammeter resistances. (c) Channel-gate voltage (*V*_ch_) dependence of *G* at *V*_bg_ = 1.5 and 3 V, corresponding to the green and black dots in panel b, respectively.

Unless stated otherwise, the measurements are performed at an electron temperature of approximately 1.3 K (Section S3). The measurement geometry is shown in Figure 1a, where *Ṽ* is the ac voltage bias and the ac current (*I*) is monitored while sweeping the back-gate (*V*_bg_) and split-gate (*V*_sg_) voltages. The result is shown in Figure 1b, where the color scale represents the measured two-terminal QPC conductance, *G*_2T_ = *I*/*Ṽ*. The dark diagonal line corresponds to the charge neutrality point of the split-gated regions, and the increase in conductance with increasing *V*_bg_ along this line is due to the formation of a QPC in between with increasing width and carrier density. We further confirm that the QPC differential conductance (*G*, obtained by subtracting the corresponding contact resistances from *G*_2T_) is quantized by fixing the (*V*_bg_, *V*_sg_) voltages at (1.5, −3.35) and (3, −5) V, corresponding to the black and green dots in Figure 1b, and sweeping the channel gate voltage (*V*_ch_) to deplete the QPC. As shown in Figure 1c, as *V*_ch_ decreases, *G* decreases in steps of 4*e* ^2^/*h*, consistent with a spin-and-valley degenerate QPC.^59^

We perform bias spectroscopy by measuring the differential conductance *G* as a function of *V*_ch_ and the dc bias voltage across the QPC (*V*_sd_ in Figure 1a, corrected by accounting for the contact resistances). The result is shown in Figure 2a for *V*_bg_ = 3 V, corresponding to a displacement field of approximately 0.5 V/nm^66^ (see Section S4 for results at *V*_bg_ = 1.5 V, showing the gate tuneability of the “0.7 anomaly” in BLG QPCs). For a clear subband visualization, the transconductance (d*G*/d*V*_ch_) is shown in Figure 2b, where the *G* plateaus appear as dark regions with approximate conductance values in 4*e* ^2^/*h* units. At *V*_ch_ ≈ −7.58 V, the lowest *G* plateau, which we call the “0.7 anomaly” structure due to its resemblance with the so-called “0.7 anomaly” (at *V*_sd_ = 0) and “0.85 anomaly” (at *V*_sd_ ≠ 0) reported in the literature,^49^ is w-shaped near *V*_sd_ = 0. At this bias, the 4*e* ^2^/*h* plateau extends from *V*_ch_ ≈ −7.45 to −6.70 V, where an x-shaped subband structure arises due to the bias and *V*_ch_ activation of the *G* = 1.5 and 2 × 4*e* ^2^/*h* QPC subbands.^80–82^ The half-plateaus, appearing at finite bias and labeled as 0.5 and 1.5, correspond to the cases where the number of occupied QPC subbands depends on the electron propagation direction^80–82^ and defines a diamond-shaped structure. The deviation of the “0.5” half-plateau conductance from 2*e* ^2^/*h* (Figure 2a,c, red horizontal lines) is a common observation in QPCs,^16,20,47,50,83^ where it is found to be around 0.25 times the full subband conductance, corresponding to ∼*e* ^2^/*h* in our case. Its origin and robustness to magnetic fields have been discussed elsewhere^83,84^ and are beyond the scope of our work. Its asymmetry with *V*_sd_ is likely due to self-gating.^47^ An additional conductance step (gray line) appears within the lowest diamond, setting the boundary between the structure resembling the “0.7 anomaly” and the 4*e* ^2^/*h* plateau. Looking back at Figure 2a, for *G* < 4*e* ^2^/*h*, one can also distinguish a conductance peak at zero *V*_sd_ or ZBP. These observations resemble the “0.7 anomaly” in GaAs-based two-dimensional electron gas systems,^16,47,85^ with *G* now being approximately 2 times larger due to the spin-and-valley degeneracy.

**Figure 2:**
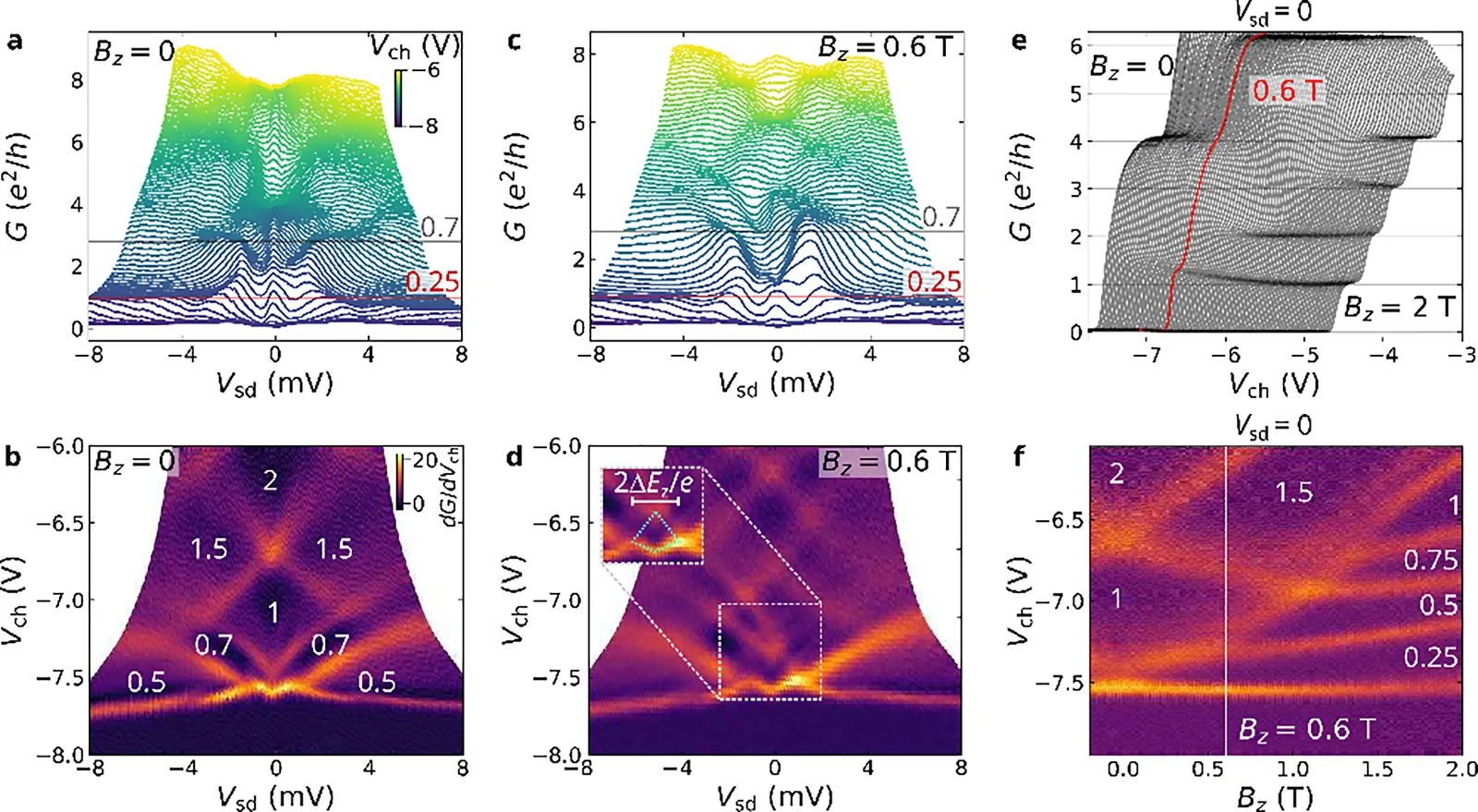
Bias spectroscopy and degeneracy lifting of the first subband at *V*_bg_ = 3 V. (a and c) Bias dependence of the differential QPC conductance at *B*_*z*_ = 0 and 0.6 T, respectively. The line color indicates *V*_ch_, as shown by the color bar in part a, and the horizontal lines at *G* = 0.7 and 0.25 × 4*e* ^2^/*h* indicate the expected position of the bias-induced plateaus labeled as 0.7 and 0.5 in part b, respectively. (b and d) Transconductance (d*G*/d*V*_ch_) obtained from parts a and c, respectively. The darker regions correspond to the conductance plateaus, as indicated by the color bar in part b. The left inset in part d shows the lowest subband, which is split by Δ*E*_Z_ ≈ 1 meV, as shown by the light-blue diamond. (e) Zero *V*_sd_ conductance vs *V*_ch_ at different *B*_*z*_ values. The curves are offset horizontally for visualization purposes, and transparency is used to highlight the trace accumulation at the plateaus. *B*_*z*_ = 0.6 T, corresponding to parts c and d, is red. (f) d*G*/d*V*_ch_ vs *B*_*z*_ and *V*_ch_ obtained from part e and colored according to the color bar in part b. The white digits in parts b and f correspond to the approximate QPC conductances in units of 4*e* ^2^/*h*.

An out-of-plane magnetic field (*B*_*z*_) causes a Berry-curvature-induced orbital effect that results in a large valley *g* factor in BLG QPCs,^61–63,86^ and a small *B*_*z*_ suffices to break the valley degeneracy in the QPC. Parts c–f of Figure 2 show that *B*_*z*_ = 0.6 T splits the lowest QPC subband into two conductance steps of approximately 1 and 3*e* ^2^/*h*. Even though the red line in Figure 2e shows that *G* ≈ 1.3*e* ^2^/*h* at 0.6 T, parts e and f of Figure 2 show that this plateau is fully developed at 1*e* ^2^/*h* for *B*_*z*_ = 2 T, indicating spin-and-valley splitting. As shown by the light-blue diamond in Figure 2d, the first subband is split by Δ*E*_Z_ ≈ 1 meV, which is larger than *E*_K_ for *T*_K_ < 10 K. Note that the spin-and-valley splitting of the lowest subband at *B*_*z*_ = 0.6 T shows an effective *g* factor of approximately 29, which may be caused by a combination of the mentioned orbital effect^61–63,86^ and exchange coupling, shown to influence *g* factors of the lowest QPC subband.^49^ In Figure 2c, near 4*e* ^2^/*h*, the ZBP acquires a strong antisymmetric component. We attribute such a shape to a Fano effect, which is compatible with Kondo physics.^87^ We thus conclude that, because the spin-and-valley-polarized subband appears at lower energy, parts c and d of Figure 2 are consistent with a lifted degeneracy Kondo state near *G* = 4*e* ^2^/*h* (see Section S6 for the detailed analysis of the *B*_*z*_ = 0.6 T data in terms of Kondo and Fano physics).

An important feature of the Kondo effect in QPCs is that *G* decreases with increasing temperature (*T*).^16,46,47^ Figure 3a shows that, at *V*_sd_ = 0 and within the gray area, *G* decreases with increasing *T* and a short plateau appears slightly below *G* = 2*e* ^2^/*h* (copper arrow), similar to a result in Si/SiGe.^50^ We have fit the *G* vs *T* data to a rescaled Kondo model:^16^ *G*_K_ = *G*_min_ + (*G*_max_ – *G*_min_)*f*_K_(*T*/*T*_K_), where *G*_max_ = 4*e* ^2^/*h*, *G*_min_ = 0.7*e* ^2^/*h* (Section S5) is the minimal QPC conductance at high temperature, and *f*_K_ = [1 + (2^1/*s*^ – 1)^2/*n*^(*T*/*T*_K_)^2^]^−*s*^ is the Kondo activation function, where *s* = 0.22 and *n* = 2 for SU(2)^7^ and *s* = 0.2 and *n* = 3 for SU(4) Kondo physics.^10^ Following ref 23, we plotted the universal Kondo scaling function $u_{K}=\sqrt{\frac{{G_{r}}^{-1/s}-1}{{\left(2^{1/s}-1\right)}^{2/n}}}$ , where $G_{r}=\frac{G-G_{\min}}{G_{\max}-G_{\min}}$ . The results are displayed in parts b and c of Figure 3 for SU(2) and SU(4), respectively. The plots show that both Kondo scalings are compatible with our *B*_*z*_ = 0 data (see Section S5 for the universal scaling of *G* vs *T*/*T*_K_). For clarity, the SU(2) and SU(4) *T*_K_ are labeled as *T*_K_^SU(2)^ and *T*_K_^SU(4)^, respectively, and plotted in Figure 3d. This figure shows that both *T*_K_’s increase with *V*_ch_. In addition, *T*_K_^SU(2)^ and *T*_K_^SU(4)^ range from values comparable to the expected SO gap^77,78^ (gray patch) and experimental resolution (black dashed line) at low *V*_ch_ to significantly greater magnitudes at less negative *V*_ch_. Because our experimental resolution is the upper bound to any spin or valley splitting, this observation suggests a transition from SU(2) to SU(4) physics. The robustness of the observation that *T*_K_^SU(2)^ is well above the upper bound for a spin or valley degeneracy lifting is supported by the inset of Figure 3b, which shows that, regardless of the choice of *G*_min_, at *V*_ch_ = −7.2 V, *T*_K_^SU(2)^ is well above 5 K. Thus, the condition required to achieve SU(4) Kondo is robust to changes in the fitting parameters (Section S5).

**Figure 3:**
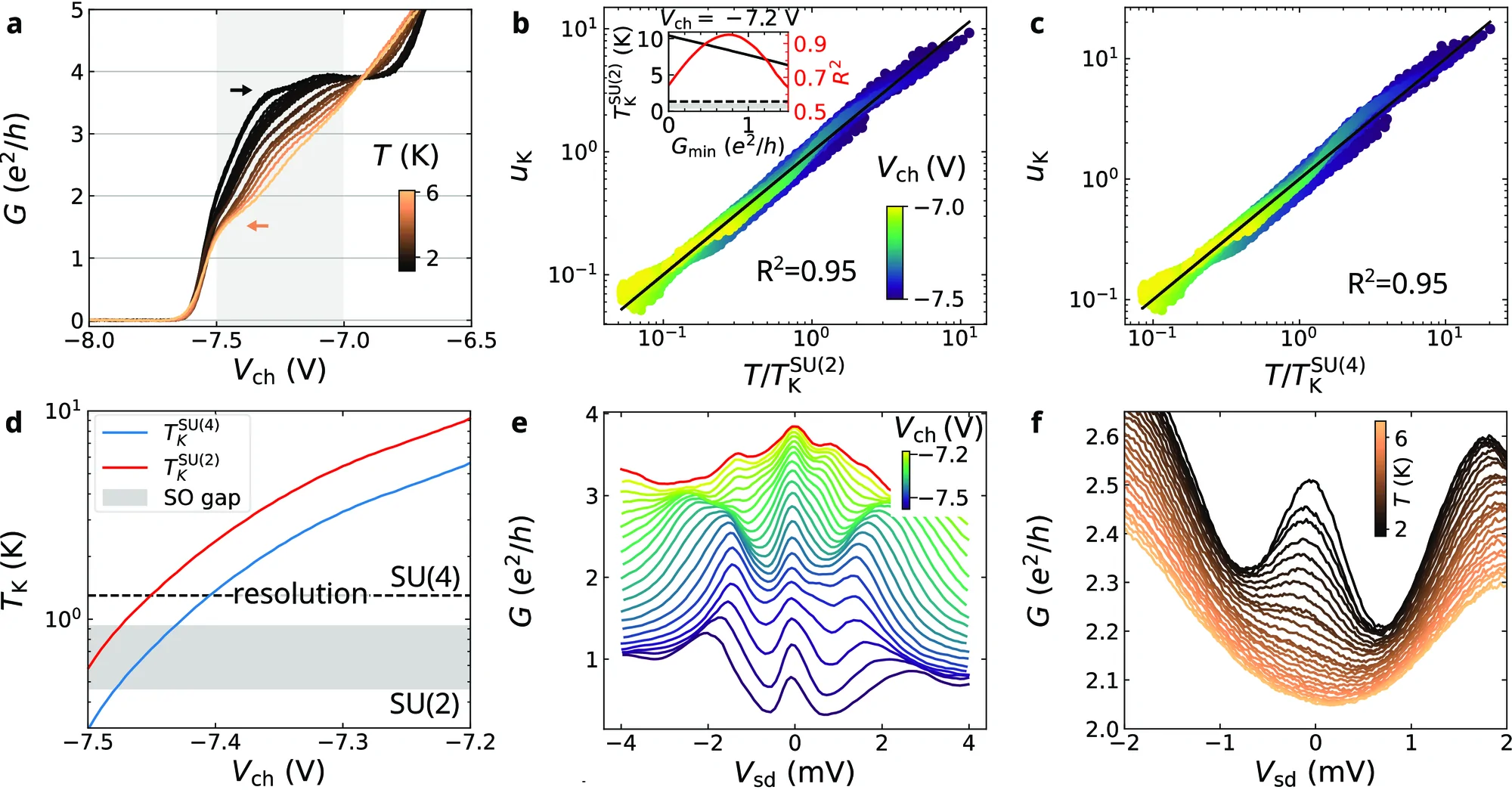
Temperature dependence of the QPC conductance at *V*_bg_ = 3 V, zero *V*_sd_, and zero applied magnetic field. (a) *V*_ch_ dependence of *G* at different temperatures, indicated by the line colors. The gray patch highlights the *V*_ch_ range used in the temperature analysis, and a small (<0.25*e* ^2^/*h*) thermally activated background conductance has been subtracted. Universal scaling of the (b) SU(2) and (c) SU(4) Kondo function *u*_K_ (see the main text) from the fits. In parts b and c, the black line is the expected theoretical value. The dots, colored according to the color bar in part b, are obtained from fits to the *G* vs *T* data from part a. The inset in part b shows the evolution of *T*_K_^SU(2)^ (*V*_ch_ = −7.2 V) and the fitting determination coefficient *R* ^2^ with *G*_min_. The black dashed line is the experimental resolution, and the gray patch at the bottom is the expected SO gap. (d) SU(2) and SU(4) *T*_K_ from the Kondo fits used to obtain parts b and c. At and below the gray patch, which represents the SO gap, SU(2) Kondo is expected. SU(4) is expected above the SO gap. The experimental resolution is shown as a black dashed line. (e) Low-bias zoom to the bias spectroscopy in Figure 2a showing the progressive widening of the ZBP. The red trace corresponds to *V*_ch_ ≈ −7.18 V, while the other traces are color-coded according to the inset color bar. (f) *T* dependence of the ZBP. The line color indicates *T*, as shown by the color bar.

The additional plateau observed in Figure 3a when *G* is slightly below 4*e* ^2^/*h* and at the lowest *T* (black arrow) resembles the effect of bound states introduced in ref 23 in a similar one-dimensional geometry. There, two small protrusions were introduced in different spots on the split-gate structure, and in addition to a plateau, a decrease in *G* below the first subband was observed for *T* > 0.2 K, an indication of the formation of a bound state. In our case, d*G*/d*V*_ch_ does not change sign at higher *T*. Due to the large aspect ratio of our QPC, this may originate from irregularities at the split-gate edges, consistent with different observations in Sections S2, S4, and S6.

The *T* dependence of the “0.7 anomaly” structure in QPCs has been analyzed using different models. The Kondo effect in “clean” QPCs, which is explained by emergent localized states in the saddle potential,^17,18,21^ results in *G*_min_ > 0.^6,17^ In contrast, the Kondo effect induced by a localized state in the QPC results in *G*_min_ = 0.^23^ Thus, the requirement of *G*_min_ > 0 to fit our data may indicate that electrostatically confined states are not central to the observed temperature dependence. We have also fit the data to the quadratic temperature dependence arising from the interpretation in terms of a smeared van Hove peak in the density of states^28,30,51^ and found that *R* ^2^ ≤ 0.8, indicating a poor fit.

The application of a *V*_sd_ breaks the Kondo state at a characteristic bias, which is determined by *E*_K_.^6,7,16^ Figure 3e is a low *V*_sd_ zoom to Figure 2a that shows the ZBPs and their apparent broadening with increasing *V*_ch_, consistent with the increasing *T*_K_ in Figure 3d. At high *V*_ch_ values (see the red trace), an oscillation emerges within the ZBP, indicating interference effects that may influence the low-bias *G* under these conditions. Independent evidence for the relatively high *T*_K_ is obtained from the *T*-dependent ZBP measured in a different cooldown and shown in Figure 3f. Its persistence beyond 2.8 K and the absence of a clear broadening indicate that the Kondo temperature lies well above 1 K.^4,8^

We now explore the effect of an in-plane magnetic field (*B*_∥_), and Figure 4a shows the *V*_ch_ dependence of the QPC conductance at different *B*_∥_ (indicated by the line color). As expected, new kinks appear at the subband locations when increasing *B*_∥_, indicating additional spin splittings. To visualize them, Figure 4b shows d*G*/d*V*_ch_ at different *B*_∥_, color-coded according to Figure 4a. One observes that, while the *G* = 2 × 4*e* ^2^/*h* subband at *B*_∥_ = 3 T is split into two (stars), the “0.7 anomaly” (squares) and the 1 × 4*e* ^2^/*h* (right triangles) subband remain separated, and an additional plateau appears between them (left triangles), resulting in three transconductance peaks, in agreement with ref 31. We attribute the latter to the spin splitting of the 4*e* ^2^/*h* subband. In Figure 4c, we characterize the QPCs bias dependence at *B*_∥_ = 3 T and confirm the presence of the new conductance level, visible as a dense accumulation of traces with finite bias that saturates approximately at 4*e* ^2^/*h*. The wing shape of this subband, which is in the range of 0.3 < |*V*_sd_| < 0.6 mV (Figure 4c, red triangles), is caused by the spin splitting of the 4*e* ^2^/*h* level. Following ref 16, we use this range to estimate the spin *g* factor, which is *g* = 2.6 ± 0.9, in agreement with the *g* = 2 obtained in graphene.^77,88^ In addition, the high *V*_sd_ tails saturating slightly above *G* = 0.7 × 4*e* ^2^/*h* remain unchanged by *B*_∥_ up to 8 T (Section S7), an indication of the robustness of the “0.7 anomaly” state at finite bias.

**Figure 4:**
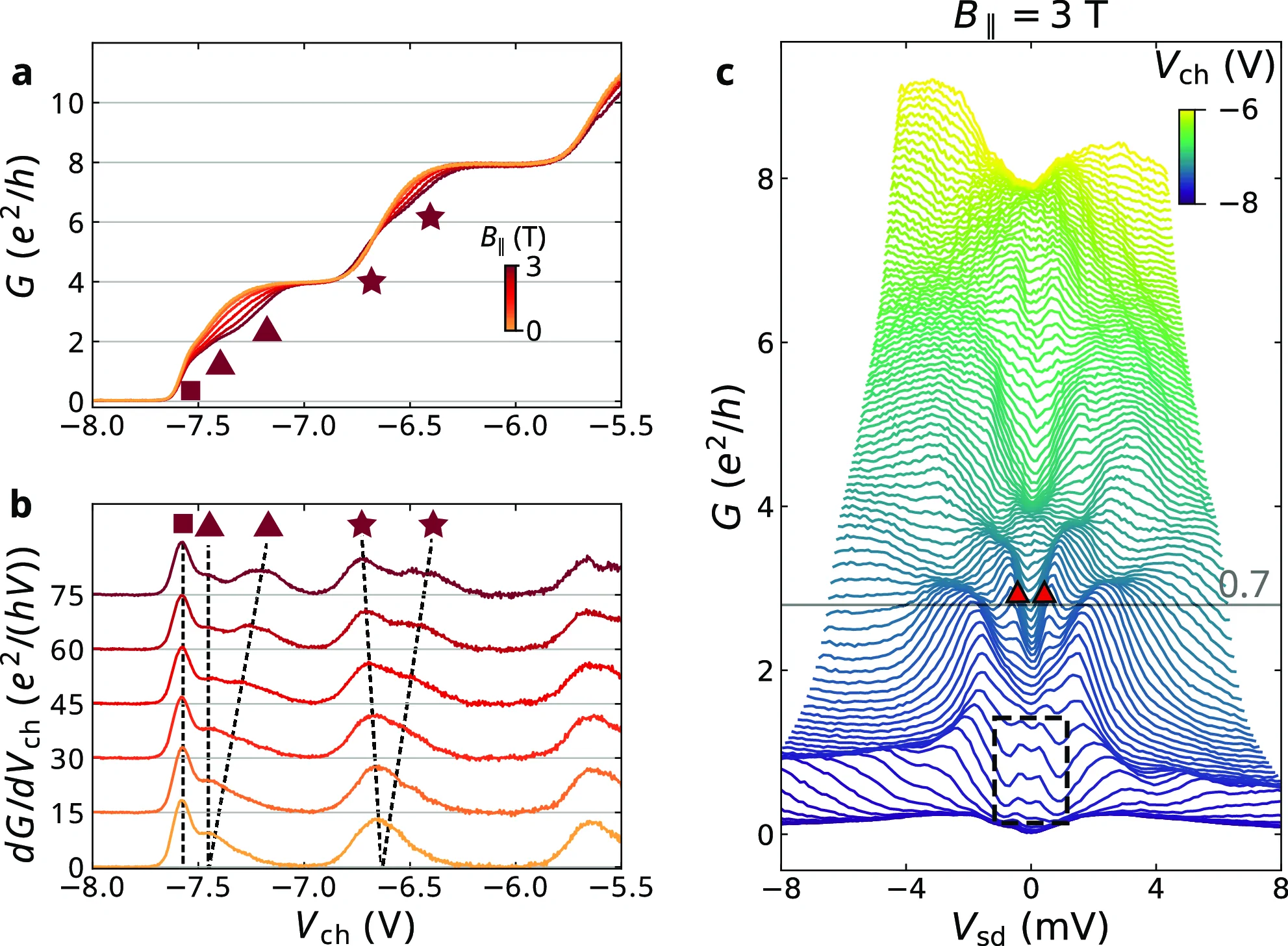
In-plane magnetic field effect at *V*_bg_ = 3 V. (a) QPC conductance and (b) transconductance vs *V*_ch_ and different *B*_∥_. The latter has been smoothed using a running average window of 15 points. The lines in both parts are color-coded according to the color bar in part a. The squares indicate the “0.7 structure”, and the triangles and stars indicate the spin splitting of the 1 and 2 × 4 *e* ^2^/*h* subbands, respectively. The dashed lines are a guide to the eye. (c) Bias dependence of the differential QPC conductance at *B*_∥_ = 3 T. The line color indicates *V*_ch_, as shown by the color bar, and the horizontal line at *G* = 0.7 × 4*e* ^2^/*h* serves as a guide to compare with Figure 2. The triangles indicate the accumulation of traces used to estimate the spin *g* factor of the 4*e* ^2^/*h* subband. The black dashed rectangle highlights the splitting of the ZBP.

Finally, we analyze the ZBPs highlighted by the dashed rectangle in Figure 4c. Their splitting is a signature of the Kondo effect.^7,16^ Assuming that the peak separation is proportional to 2g*μ_B_ *B*, we obtain g* = 2 ± 0.5, consistent with the splitting of a spin ^1^/_2_ electronic state responsible for the Kondo effect.^5^ However, for *G* higher than 2*e* ^2^/*h* and temperatures above 1.7 K, we find that the ZBP disappears with *B*_∥_ (Section S9). Such a ZBP behavior is consistent with previous reports on GaAs/AlGaAs QPCs.^16,27^

To conclude, we have performed transport experiments on a BLG-based QPC, showing features consistent with the Kondo effect. These include universal scaling of *G* vs *T*, a ZBP that splits under *B*_∥_ and has a width that scales like *T*_K_, and a faint plateau around 2*e* ^2^/*h* at zero bias, which increases above 0.7 × 4*e* ^2^/*h* with increasing bias, resembling the “0.7 anomaly”. Because *E*_K_ spans values comparable to the SO gap and extends to more than 4 times larger than any possible spin or valley splitting, we conclude that conventional SU(2) Kondo scaling should not suffice to explain these results, and SU(4) Kondo, which also fits our data, may be at play for the less negative *V*_ch_ values used in the experiment. In addition, we break the degeneracy by applying an out-of-plane magnetic field and show signatures of Kondo effects in this regime with similar *T*_K_, an indication of the robustness and tunability of the effect. We expect that these results will trigger further theoretical and experimental efforts required to establish the role of spin-and-valley degeneracy on the Kondo-like features of the “0.7 anomaly” in BLG QPCs.

## Supplementary Material



## Data Availability

All of the data and the code associated with the analysis are available in ref 90.

## Notes

Data analysis workflow, device geometry, electronic measurements, QPC formation at different electric fields, Kondo analysis and *G*_min_ dependence, temperature-dependent transport with *B*_*z*_ = 0.6 T and Fano physics, in-plane magnetic field dependence in a different cooldown, temperature and magnetic-field dependence of the ZBP, determination of the valley *g* factor, and supplementary Figures S1–S12 (PDF)
