# Supplementary material for: Tunable Kondo Behavior in a Bilayer Graphene Quantum Channel
Source: Nano Lett. 2026 Sep 16;26(38):12620–7. doi: 10.1021/acs.nanolett.6c01707 (PMC13632592; doi:10.1021/acs.nanolett.6c01707)
Supplement: Supplementary file 1 [file nl6c01707_si_001.pdf]

# Supplementary Information of 'Tunable Kondo behavior in a bilayer graphene quantum channel'

Josep Ingla-Aynés,[1,*] Serhii Volosheniuk,[1] Talieh S. Ghiasi,[1] Angelika Knothe,[2] Kenji Watanabe,[3] Takashi Taniguchi,[4] Vladimir I. Fal'ko,[5,6] and Herre S. J. van der Zant[1]

[1] *Kavli Institute of Nanoscience, Delft University of Technology, Lorentzweg 1, 2628 CJ Delft, The Netherlands*

[2] *Institut für Theoretische Physik, Universität Regensburg, D-93040 Regensburg, Germany*

[3] *Research Center for Functional Materials, National Institute for Materials Science, 1-1 Namiki, Tsukuba 305-0044, Japan*

[4] *International Center for Materials Nanoarchitectonics, National Institute for Materials Science, 1-1 Namiki, Tsukuba 305-0044, Japan*

[5] *National Graphene Institute, University of Manchester, Booth St. E. Manchester, M13 9PL, United Kingdom*

[6] *Department of Physics and Astronomy, University of Manchester, Oxford Road, Manchester, M13 9PL, United Kingdom*

(Dated: August 27, 2026)

**CONTENTS**


S1. Data analysis workflow 2

S2. Device geometry 3

S3. Electronic measurements 4

S4. QPC formation at different electric fields 5

S5. Kondo analysis and $G_{\mathrm{min}}$ dependence 6

S6. Temperature dependent transport with $B_z = 0.6$ T and Fano physics 9

S7. In-plane magnetic field dependence in a different cooldown 11

S8. Temperature dependence of zero-bias peak 12

S9. Magnetic field dependence of zero-bias peak 13

S10. Determination of the valley $g$ factor 14

References 16


## S1. DATA ANALYSIS WORKFLOW

Figure S1 displays a diagram of the workflow followed to infer the physical origin of the observed features. The observation of $4e^2/h$ conductance steps at zero magnetic field ($B$) implies that any degeneracy lifting is smaller than our experimental resolution, which is


* jingla@mit.edu

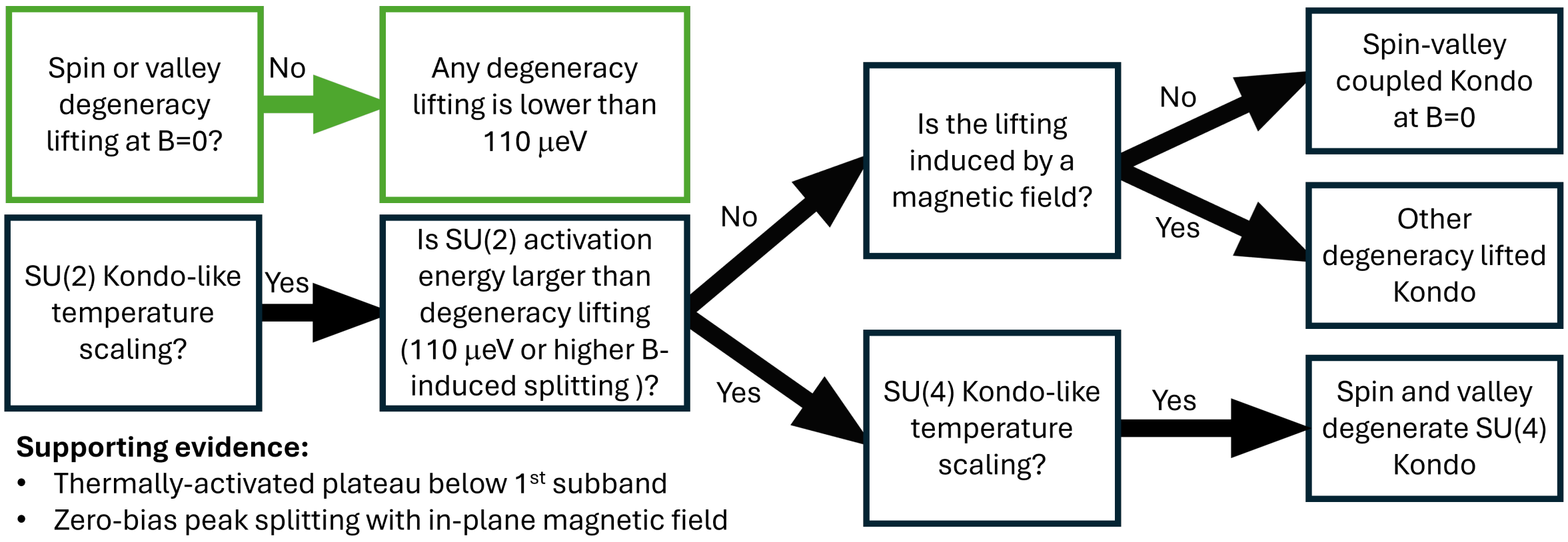


Figure S1. Diagram describing the data analysis workflow in the main manuscript.

110 $\mu$eV. The agreement between SU(2) Kondo-like temperature scaling and the data, together with the observation of a zero bias peak and its splitting with in-plane magnetic field, supports the idea that the Kondo effect is responsible for the observed structure [1]. When the activation energies exceed any possible degeneracy lifting, the Kondo effect is expected to be spin- and valley-degenerate. This observation, together with the consistency of the temperature dependence with the SU(4) model, supports an interpretation in terms of SU(4) Kondo scaling. When the activation energy is lower or comparable to 110 $\mu$eV, at $B = 0$, it is expected that the Kondo effect couples electrons with different spin and valley. Applying $B$, such as in Figs. 2 and 4 of the main manuscript, other degeneracy liftings occur, resulting in a Kondo effect with lower degeneracy.

## S2. DEVICE GEOMETRY

Figure S2a is a sketch of the measured device, and Fig. S2b shows an atomic force microscopy image taken before depositing the $Al_2O_3$. The image shows imperfections at the split gate edges, which are consistent with our observations in the main manuscript and Sections S3 and S5.

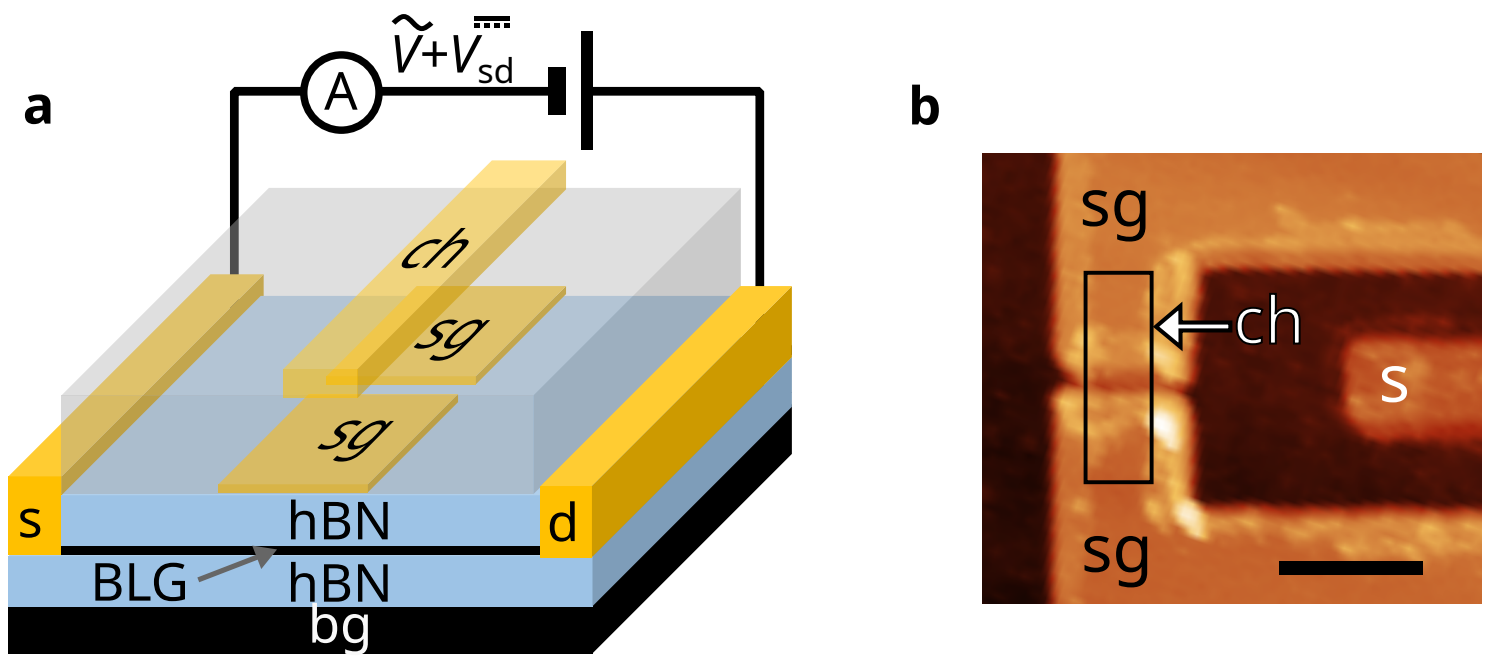


Figure S2. (a) Schematic of the measured device where the graphite back gate (bg) and bilayer graphene (BLG) are black, the hexagonal boron nitride (hBN) flakes are light blue, the Ti/Au split gates (sg), the channel gate (ch), and the source (s) and drain (d) contacts are golden, and the $Al_2O_3$ layer under ch is semi-transparent. (b) Atomic force microscopy of the device before channel gate preparation. Its approximate position is indicated by the rectangle. The scale bar is 0.6 $\mu$m.

## S3. ELECTRONIC MEASUREMENTS

To obtain clean data, we applied an ac voltage $\tilde{V} = 100$ $\mu$V to our sample. To estimate the electron temperature ($T$), we have used $T = \sqrt{T_l^2 + (e\tilde{V}/k_B)^2}$, where $T_l$ is the lattice temperature, $e$ is the electron charge, $k_B$ is the Boltzmann constant, and $e\tilde{V}/k_B \approx$1.16 K. The validity of this approximation is confirmed by the fact that the temperature-dependent analysis yields very similar results when the data obtained at the lowest temperatures are not used. In particular, we have performed the Kondo analysis excluding the data below 2 K and obtained similar results as reported in the main manuscript, with Kondo temperatures exceeding 5 K and $R^2$ values up to 0.91, supporting the robustness of our analysis.

In the measurement circuit, which is shown in Fig. S2a, one can see that the resistances of the electrodes, the BLG channel, and the ammeter are also measured and need to be subtracted to obtain the conductance ($G$) of the quantum point contact (QPC). The ammeter resistance, which was measured separately, is 3 k$\Omega$. The contact resistances depend on the backgate voltage ($V_{bg}$), and are 377 $\Omega$ and 570 $\Omega$ at $V_{bg}$ = 3 and 1.5 V, respectively. These values are, respectively, 100 $\Omega$ and 250 $\Omega$ larger than the values obtained by setting the split gate voltage to zero and were extracted by setting the lowest QPC subband to $G = 4e^2/h$, where $e$ is the electron charge and $h$ the Plank constant. We attribute these small differences to

the effect of QPC areas not covered by the channel gate. Note that the contact resistances are significantly smaller than typical QPC resistances ($h/4e^2 \approx 6.4\,\mathrm{k\Omega}$) in our experiment.

## S4. QPC FORMATION AT DIFFERENT ELECTRIC FIELDS

Figure S3 shows the dc bias ($V_{\mathrm{sd}}$) and channel gate voltage ($V_{\mathrm{ch}}$) dependence of $G$ at a back gate voltage $V_{\mathrm{bg}} = 3$ and 1.5 V. The zero-bias peak (ZBP) height at low $G$ is less pronounced at $V_{\mathrm{bg}} = 1.5$ V than at 3 V. Looking at Fig. S3d, one can also observe that, at zero $V_{\mathrm{sd}}$, the separation between the 0.7 and $1{\times}4e^2/h$ subbands is more pronounced than in Fig. S3b. Finally, a zigzag pattern is observed in the transconductance plot at $V_{\mathrm{bg}} = 1.5$ V for low $V_{\mathrm{sd}}$ and below the $4e^2/h$ subband. We attribute this structure to faint Coulomb diamonds. They indicate that weakly confined quantum dots may form within the QPC. Since the potential inside BLG QPCs is not flat [2], its depth at the center depends on the width. This implies that having a varying QPC width due to rough split gate edges may result in weakly confined areas in the QPC under certain conditions.

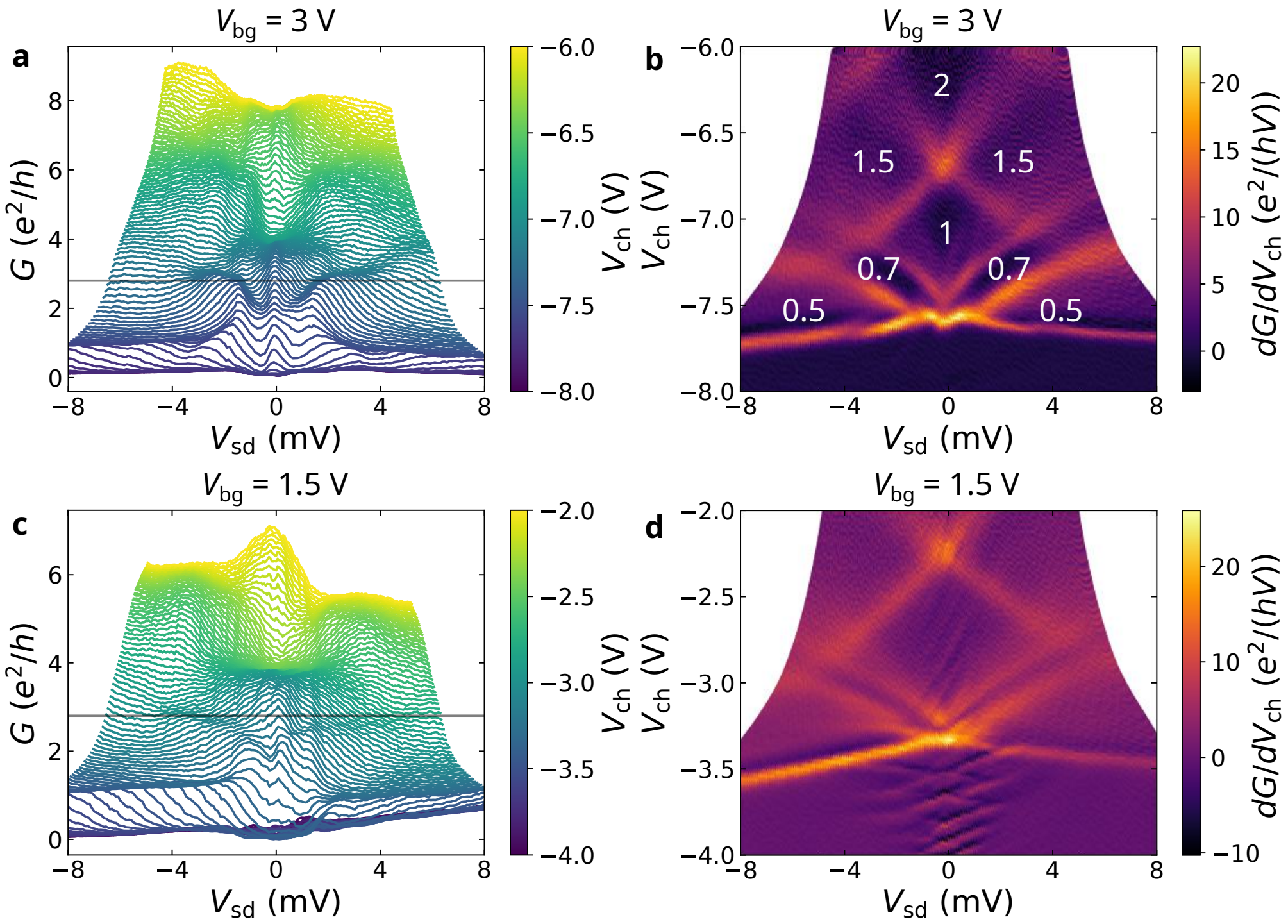


Figure S3. (a) and (c) Bias ($V_{sd}$)-dependence of the QPC conductance ($G$) at different channel gate voltages ($V_{ch}$), indicated by the line colors. The gray lines show $G = 0.7 \times 4e^2/h$, where the 0.7 anomaly is expected. (b) and (d) Transconductance $dG/dV_{ch}$ vs. $V_{sd}$ and $V_{ch}$ obtained from panels a and c, respectively.

## S5. KONDO ANALYSIS AND $G_{\mathrm{min}}$ DEPENDENCE

According to the Kondo model used here [1], the QPC conductance follows

$$G_{\mathrm{K}} = G_{\mathrm{min}} + (G_{\mathrm{max}} - G_{\mathrm{min}}) f_{\mathrm{K}}(T/T_{\mathrm{K}}), \tag{1}$$

where $G_{\mathrm{max}} = 4e^2/h$, $G_{\mathrm{min}}$ is the minimal QPC conductance at high temperature, $f_{\mathrm{K}} = [1 + (2^{1/s} - 1)^{2/n}(T/T_{\mathrm{K}})^2]^{-s}$ is the Kondo activation function, $T_{\mathrm{K}}$ the Kondo temperature, $s = 0.22[0.2]$, and $n = 2[3]$, corresponding to SU(2)[SU(4)] Kondo [1, 3]. In the manuscript, we show the universal Kondo scaling function, which is defined as:

$$u_{\mathrm{K}} = \sqrt{\frac{{G_{\mathrm{r}}}^{-1/s} - 1}{(2^{1/s} - 1)^{2/n}}}, \tag{2}$$

where $G_{\mathrm{r}} = \frac{G - G_{\mathrm{min}}}{G_{\mathrm{max}} - G_{\mathrm{min}}}$. Note that, in the ideal case $G_{\mathrm{r}} = f_{\mathrm{K}}$ and $u_{\mathrm{K}} = T/T_{\mathrm{K}}$.

The fitting procedure is illustrated in Fig. S4, where the raw measured data are plotted in panel a, and the results of the Kondo analysis are shown.

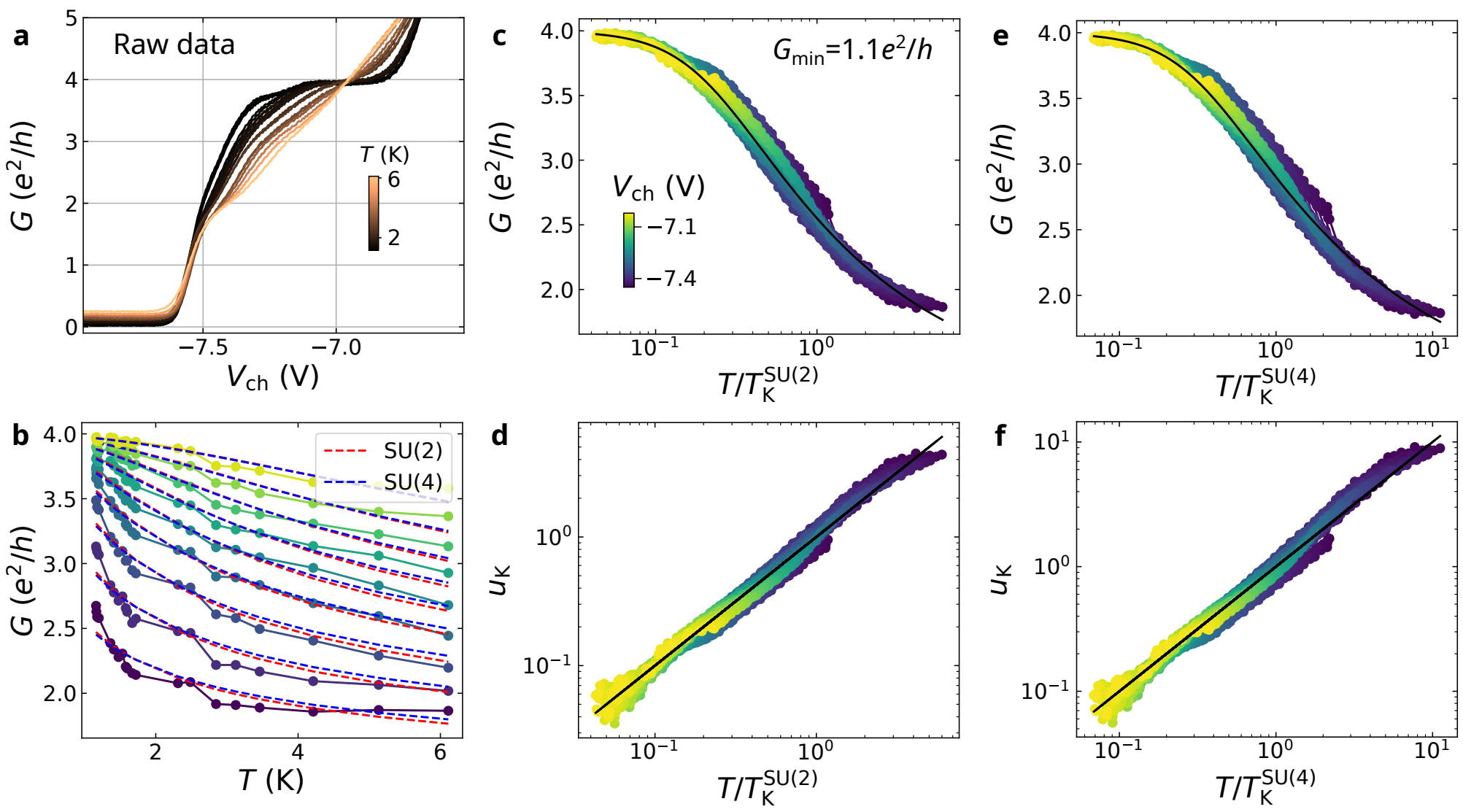


Figure S4. (a) $G$ vs. $V_{\mathrm{ch}}$ at different temperatures ($T$) indicated by the line color. (b) $G$ vs. $T$ extracted from panel a. The dots correspond to experimental data and the dashed lines to the fits to SU(2) and SU(4) Kondo models. (c) and (e) $G$ vs. normalized temperature ($T/T_{\mathrm{K}}$) for SU(2) and SU(4) Kondo models, respectively. (d) and (f) Universal Kondo scaling function $u_{\mathrm{K}}$ vs. $T/T_{\mathrm{K}}$ for SU(2) and SU(4) Kondo models, respectively. $G_{\mathrm{min}}$ is the same in both models. The coefficients of determination for both SU(2) and SU(4) fits are $R^2 = 0.95$.

A close look at Fig. S4a at low $G$ indicates that the pinch-off region ($G < 0.5e^2/h$) acquires a finite conductance when increasing $T$. This result indicates the presence of a thermally activated path that is independent of $V_{\mathrm{ch}}$, and likely lies underneath the split gates, outside the QPC area. For this reason, we have subtracted the contribution of such a parallel channel assuming its $G$ is the one measured in the $V_{\mathrm{ch}}$ range between $-8$ and $-7.75$ V when performing the Kondo analysis, as shown in Fig. S5.

Given that $G_{\mathrm{min}}$ is the only free parameter besides $T_{\mathrm{K}}$, one might question the robustness of the observed universal scaling. An important observation of our work is that the SU(2) Kondo temperature $T_{\mathrm{K}}^{\mathrm{SU(2)}}$ becomes large enough to indicate a transition between SU(2) and SU(4) Kondo physics. For this reason, we have analyzed the robustness of the fits by performing SU(2) Kondo fits for different values of $G_{\mathrm{min}}$ and used the coefficient of determination ($R^2$) as an indication of the goodness of the fit. In Fig. S6, we represent $R^2$ and $T_{\mathrm{K}}^{\mathrm{SU(2)}}$ at $V_{\mathrm{ch}} = -7.2$ V as a function of $G_{\mathrm{min}}$ and compare the results for corrected

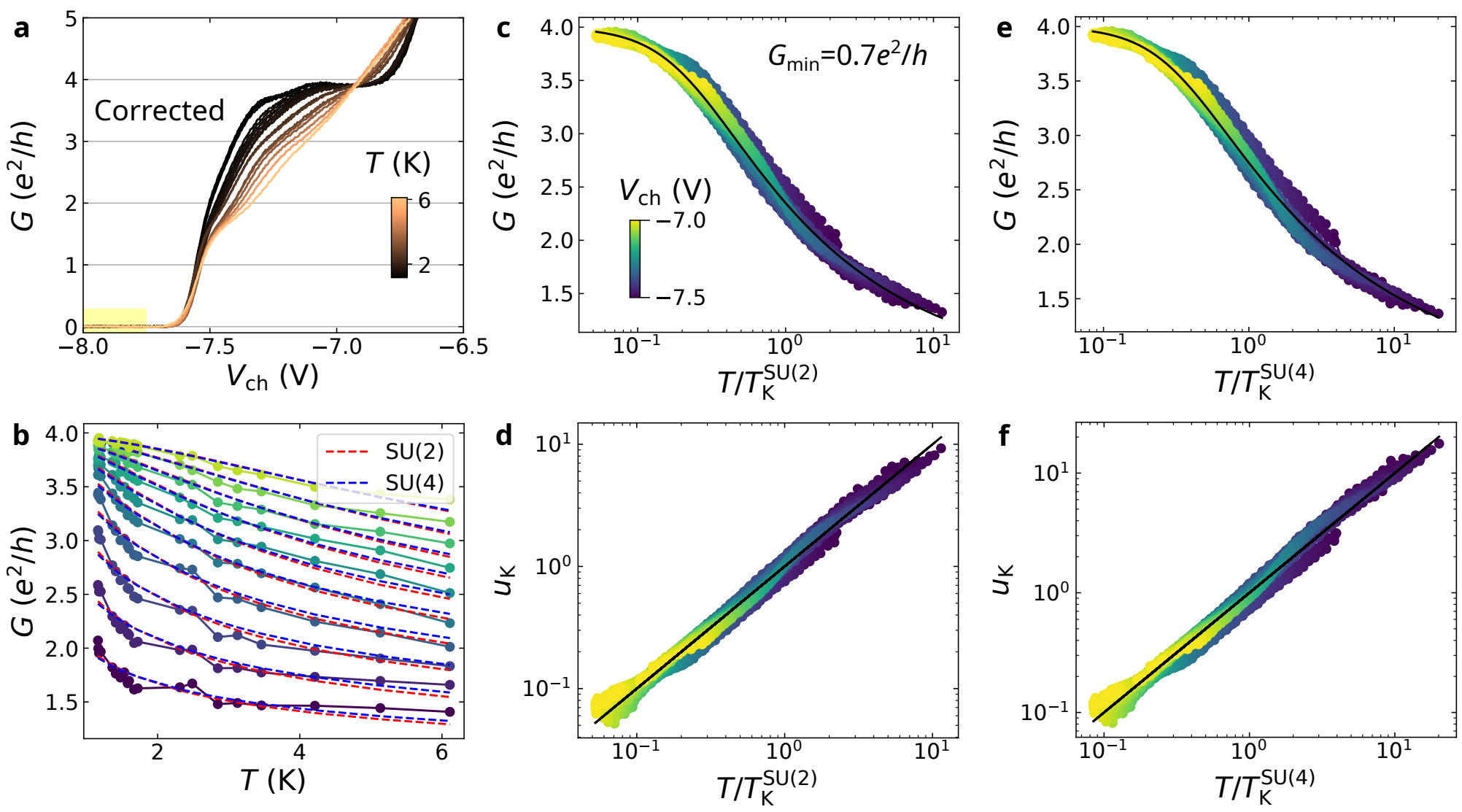


Figure S5. (a) $G$ vs. $V_{\rm ch}$ at different temperatures ($T$) indicated by the line color. The yellow stripe indicates the region used to correct for the parallel, thermally-activated conductance path. (b) $G$ vs. $T$ extracted from panel a. The dots correspond to experimental data and the dashed lines to the fits to SU(2) and SU(4) Kondo models. (c) and (e) $G$ vs. normalized temperature ($T/T_{\rm K}$) for SU(2) and SU(4) Kondo models, respectively. (d) and (f) Universal Kondo scaling function $u_{\rm K}$ vs. $T/T_{\rm K}$ for SU(2) and SU(4) Kondo models, respectively. $G_{\rm min}$ is the same in both models.

(Fig. S5a) and non corrected (Fig. S4a) data.

Figure S6 confirms that, regardless of the choice of $G_{\rm min}$ and whether the thermally activated parallel channel is corrected for, $T_{\rm K}^{\rm SU(2)}(V_{\rm ch}\text{=-7.2 }V) \gtrsim 5$ K, at least four times greater than any possible spin or valley splitting, as indicated by the black dashed line. We note that, in both panels $G_{\rm min}$ increases above the minimal experimental conductance values, resulting in poor fits and low $R^2$. For this reason, the most likely $T_{\rm K}^{\rm SU(2)}$ corresponds to $G_{\rm min}$ producing a maximum $R^2$, which is approximately 0.7 when corrected and $1.1 \times e^2/h$ when analyzing the raw data (Fig. S6b), resulting in $T_{\rm K}^{\rm SU(2)} = 8.5$ and 9 K, respectively.

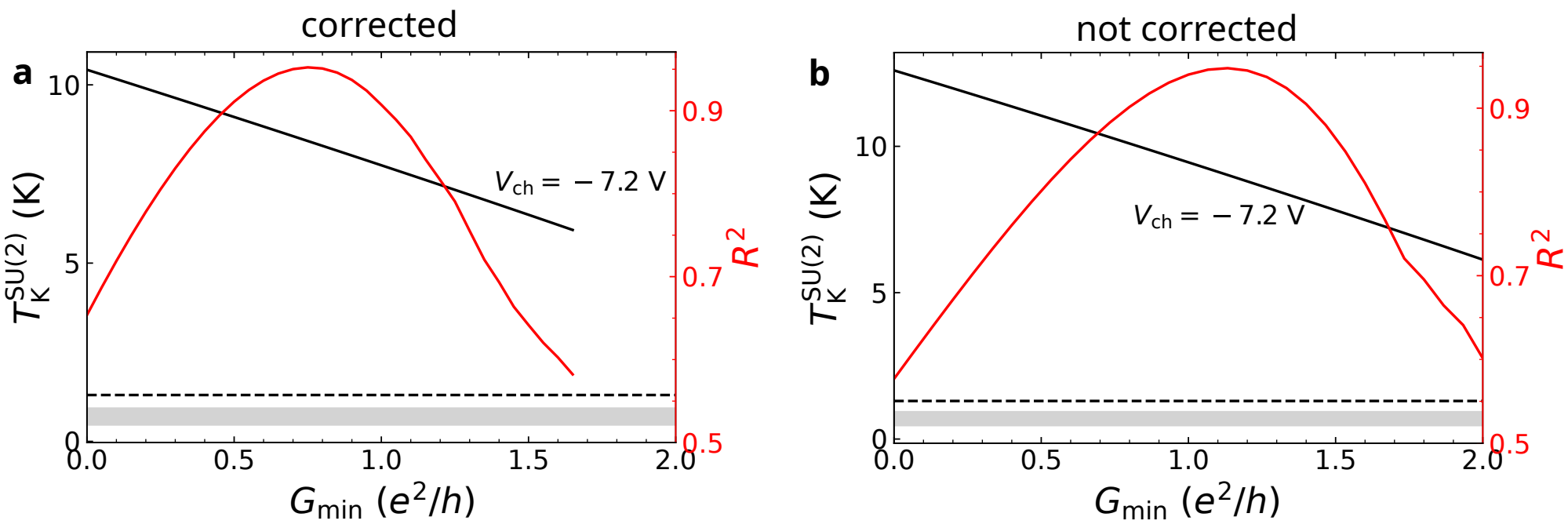


Figure S6. $T_K^{SU(2)}(V_{ch} = -7.2\text{ V})$ and coefficient of determination $R^2$ vs. $G_{min}$. (a) shows the result for the data corrected as in Fig. S5a and (b) for uncorrected data (Fig. S4a). The gray patch represents the spin-orbit coupling from the literature and the black dashed lines the experimental resolution, which is the upper bound to any spin or valley splitting at zero magnetic field.

## S6. TEMPERATURE DEPENDENT TRANSPORT WITH $B_z = 0.6$ T AND FANO PHYSICS

The temperature dependence of the zero-bias $G$ under an out-of-plane magnetic field $B_z = 0.6$ T is shown in Fig. S7a, where the yellow patch highlights the $V_{ch}$ range that shows Kondo-like thermal activation ($G$ decreases when increasing $T$). Figure S7b shows the $V_{sd}$ dependence, which is also plotted in Fig. 2c of the main manuscript, and the relevant area is marked in yellow to highlight the asymmetric Fano-like ZBP structure. Figure S7c shows the universal Kondo scaling extracted using Equations 1 and 2. It is worth noting that, for each $V_{ch}$, a single fitting parameter $T_K$ is used, whereas a single value of $G_{min}$=2.2$e^2/h$ is fixed for all the $V_{ch}$ values, making $R^2 = 0.93$ a significant value. The enhanced $G_{min}$ with respect to the $B_z = 0$ case indicates a change in the degeneracy of the Kondo state. However, the Kondo temperature range (approximately 1 to 13 K) remains similar to the $B_z = 0$ case.

To infer whether the bias dependence is indeed consistent with Fano physics, we have fit the $V_{ch}$ dependence to the Fano formula [4]:

$$G(V_{sd}) = G_0 + c_1 V_{sd} + A\frac{(\epsilon + q)^2}{(1 + \epsilon^2)} \tag{3}$$

where $G_0$ and $C_1$ are the background conductance and slope, $A$ is the resonance strength, $q$ is the asymmetry factor, $\epsilon = (eV_{sd} - E_0)/\Gamma$, where $e$ is the electron charge, $E_0$ and $\Gamma$ are

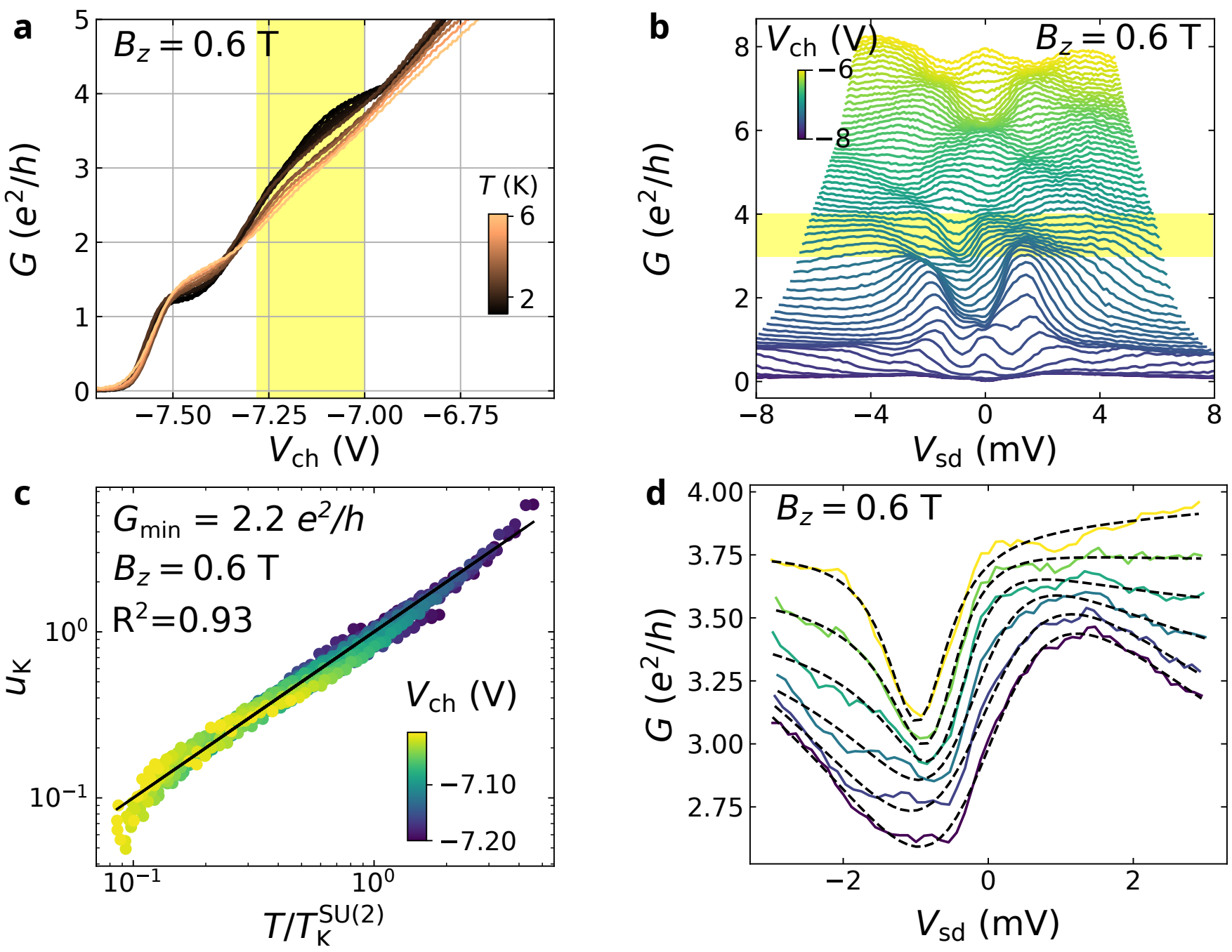


Figure S7. (a) Temperature dependent $G$ vs. $V_{ch}$. The yellow patch indicates the area showing the Kondo-like thermal activation. The data has been corrected as Fig. S5a (b) Bias and channel gate dependence with the yellow patch as in a, where the asymmetric Fano-like structure near zero $V_{sd}$ can be observed. (c) SU(2) Kondo universal scaling with $G_{min} = 2.2e^2/h$. (d) Fitting of the low $V_{sd}$ conductance using Eq. 3. The lines colored according to the colorbar in c are the extracted from panel b. The fits are black dashed lines.

the resonance energy and linewidth. The fits are shown in Fig. S7d as black dashed lines, and the parameters obtained from the fits are shown in Table S1. From these, we conclude that, except for the $V_{ch} = -7.042$ V case, which has a negative $\Gamma$ that is likely an artifact of the fit, the remaining traces are consistent with Fano physics. The faint Coulomb diamonds in Fig. S3d, together with the plateau at $G \approx 0.9 \times 4e^2/h$ in Fig. 3a suggest that variations in the effective QPC width may lead to weakly confined regions when $B_z$ induces magnetic depopulation [5]. Under such conditions, interference effects consistent with Fano physics [6] provide a plausible explanation for the observed asymmetric ZBPs.

| $G_0$ (e$^2$/h) | $c_1$ (e$^2$/hV) | $A$ (e$^2$/h) | $E_0$ (eV) | $\Gamma$ (eV) | $q$ | $V_{ch}$ (V) |
|---|---|---|---|---|---|---|
| 3.637(24) | $-137.5(11)$ | $-0.421(28)$ | $-1.37(35) \times 10^{-4}$ | $1.40(5) \times 10^{-3}$ | $-1.35(5)$ | -7.192 |
| 3.678(31) | $-119.6(15)$ | $-0.389(36)$ | $-2.26(52) \times 10^{-4}$ | $1.41(8) \times 10^{-3}$ | $-1.34(7)$ | -7.162 |
| 3.640(20) | $-49.4(11)$ | $-0.267(24)$ | $-3.25(47) \times 10^{-4}$ | $1.02(7) \times 10^{-3}$ | $-1.46(9)$ | -7.132 |
| 3.660(11) | $-9.46(56)$ | $-0.147(13)$ | $-5.20(28) \times 10^{-4}$ | $6.86(34) \times 10^{-4}$ | $-2.01(10)$ | -7.101 |
| 3.734(8) | 4.41(38) | $-0.0561(65)$ | $-7.24(18) \times 10^{-4}$ | $5.75(21) \times 10^{-4}$ | $-3.48(21)$ | -7.072 |
| 3.853(9) | 22.91(52) | $-0.0010(14)$ | $-9.61(26) \times 10^{-4}$ | $-5.21(27) \times 10^{-4}$ | 26.8(186) | -7.042 |

Table S1. Fano fit parameters reported with uncertainties in a value(uncertainty) format.

## S7. IN-PLANE MAGNETIC FIELD DEPENDENCE IN A DIFFERENT COOLDOWN

To achieve higher in-plane magnetic field values $B_\parallel$, we loaded the sample in a cryostat with a base temperature $T \approx 1.7$ K and $-8 < B_\parallel < 8$ T. The results are shown in Fig. S8 and show the high bias subband at $G \approx 0.7 \times 4e^2/h$ and the ZBP observed in the first cooldown at $T \approx 1.2$ K, which here has a reduced height. Furthermore, the spin splitting of the $4e^2/h$ plateau at $B_\parallel$= 3 T is also reproduced, and the formation of a well-defined plateau at $G \approx 2e^2/h$ is observed at $B_\parallel$= 8 T. The corresponding transconductance plots are shown in Figs. S8d-S8f.

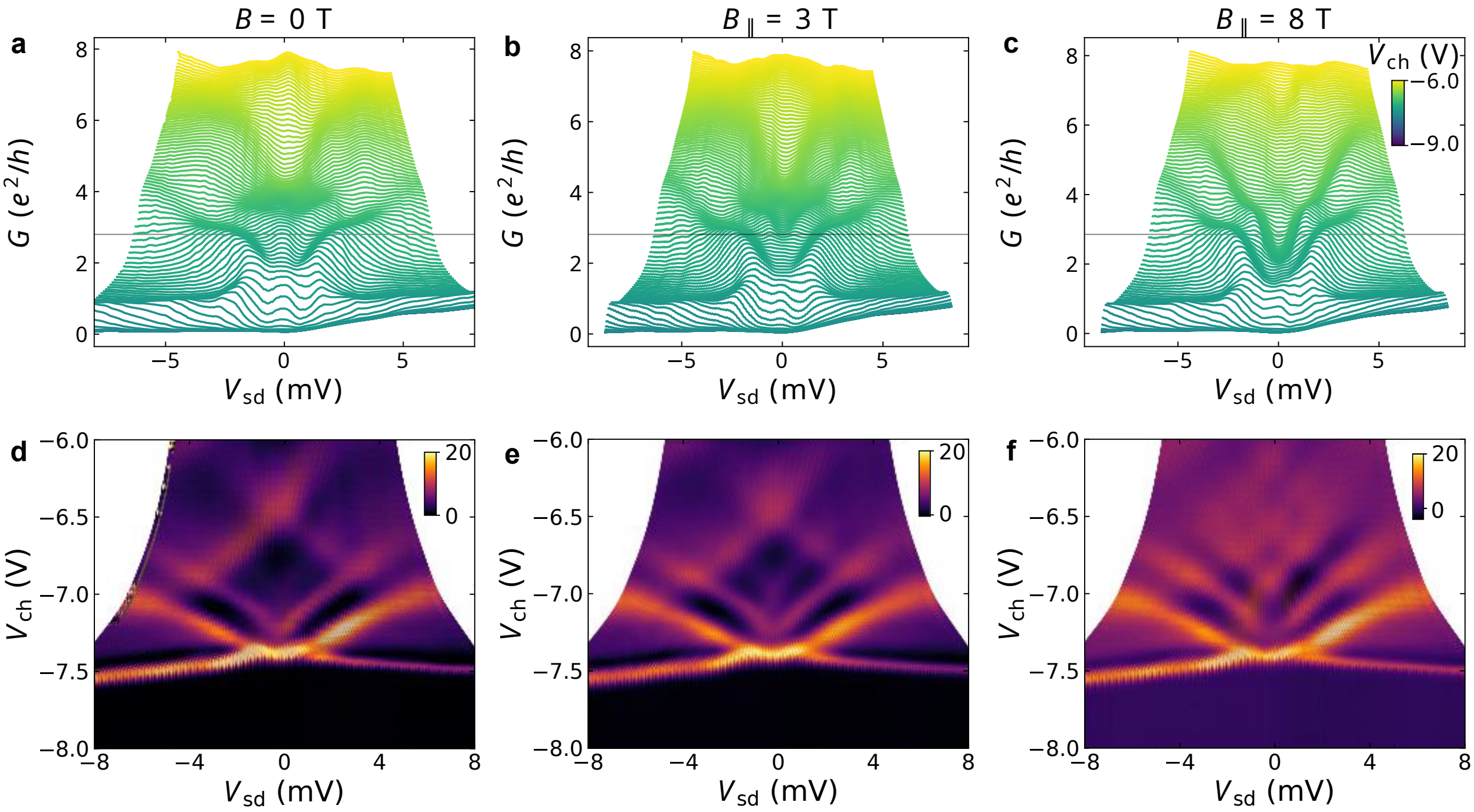


Figure S8. (a)-(c) Evolution of $G$ vs. $V_{\rm sd}$ at different $V_{\rm ch}$ with $B_{\parallel}$ at $T \approx 1.7$ K and $V_{\rm bg} = 3$ V. The titles indicate the $B_{\parallel}$ values, $V_{\rm ch}$ is shown by the line colors, defined by the color bar in panel c, and the horizontal gray lines show $G = 0.7 \times 4e^2/h$. (d)-(f) Transconductance extracted from panels a-c, respectively.

## S8. TEMPERATURE DEPENDENCE OF ZERO-BIAS PEAK

The $T$ evolution of the ZBPs is shown in Fig. S9 for different $V_{\rm ch}$ and shows that, for $V_{\rm ch} \leq -7.2$ V, the peak disappears when heating. In contrast, for $V_{\rm ch} = -7$ V, instead of a single peak, two peaks are observed at 1.7 K. When heating, a single peak arises near $V_{\rm sd} = 0$. We attribute these behaviors to the complex $T$ dependence shown by the 0.7 anomaly in QPCs [7]. Note that, for $V_{\rm ch} < -7.2$ V, the zero bias $G$ increases with $T$, indicating a thermally activated current path, most likely under the split gates due to the small gap.

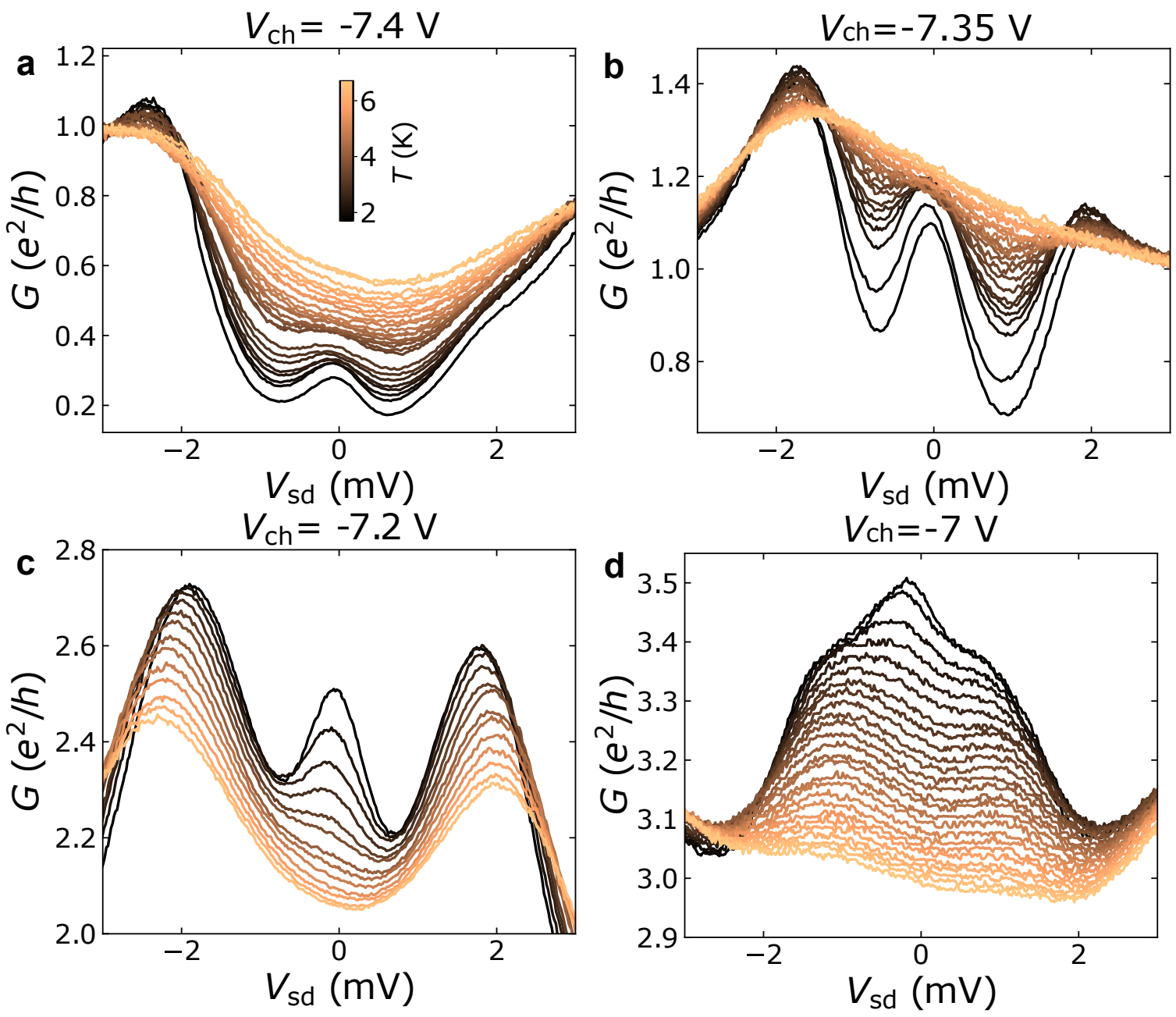


Figure S9. Zero bias peak evolution with temperature at $V_{ch} = -7.4$ (a), $V_{ch} = -7.35$ (b), $V_{ch} = -7.2$ (c), and $V_{ch} = -7$ V (d). The line color, specified by the color bar in panel a, shows the temperature $T$. These results were obtained during the second cooldown at $B = 0$ and $T \approx 1.7$ K.

## S9. MAGNETIC FIELD DEPENDENCE OF ZERO-BIAS PEAK

The in-plane magnetic field dependence of the ZBP measured at $V_{ch} = -7.2$ V is shown in Fig. S10. In contrast with Fig. 3 in the main manuscript, the zero bias peak does not split into two when increasing $B_{\|}$ and instead turns into a minimum. This behavior has been observed at other $V_{ch}$ in Fig. 3 of the main manuscript and in previous QPC works in 2DEG systems, such as Refs. [1, 7].

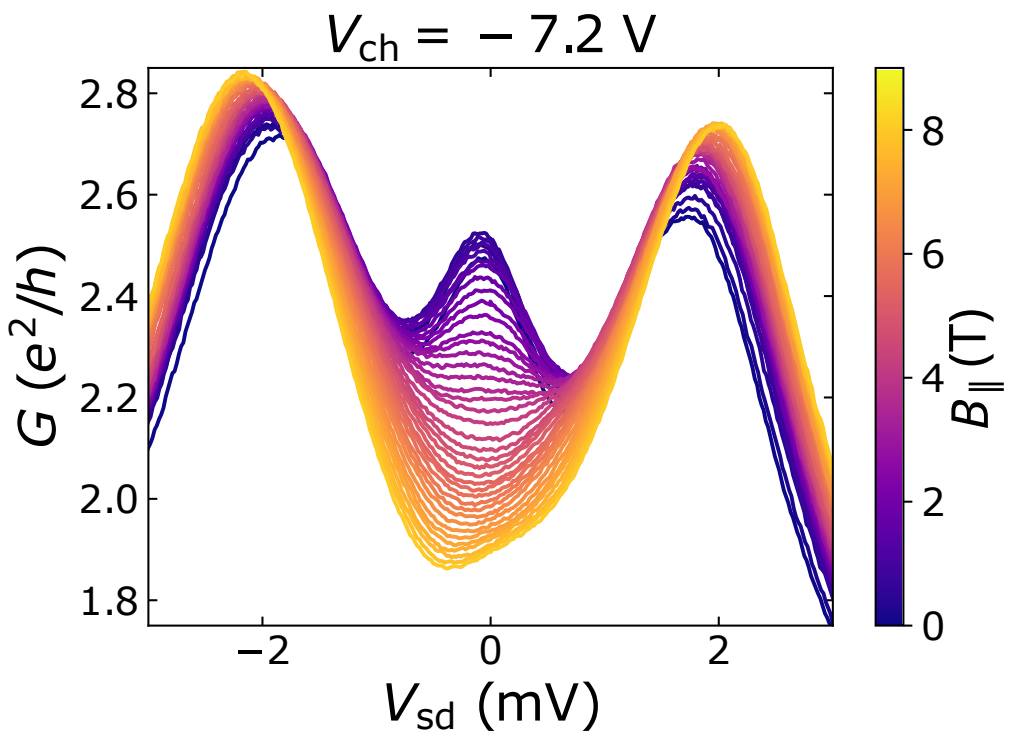


Figure S10. In-plane magnetic field dependence of the zero bias peak at $T \approx 1.7$ K and $V_{ch} = -7.2$ V. These results were obtained during the second cooldown at $T \approx 1.7$ K.

## S10. DETERMINATION OF THE VALLEY $g$ FACTOR

It has been shown that BLG QPCs respond to an out-of-plane magnetic field $B_z$ with a valley splitting [8–10]. The energy splitting is proportional to the applied $B_z$ and follows $\Delta E_Z = g_v \mu_B B_z$, where $g_v$ is the valley $g$ factor and $\mu_B$ is the Bohr magneton. Since $g_v$ depends on the QPC width [11], we have measured the effect of $B_z$ on the QPC conductance and transconductance to determine $g_v$. The results are shown in Fig. S11. In panels a-c, the $G$ traces are shown vs. $V_{sd}$ and for different $V_{ch}$, demonstrating the breaking of the subband degeneracy from 4 down to 2, due to the valley splitting. Figure S11d-f shows the QPC transconductance, which we used to extract the valley splitting $\Delta E_z$. The white horizontal lines show $2\Delta E_z/e$ for the $G = 2 \times 4e^2/h$ subband. The $\Delta E_z$ values have been collected and plotted vs. $B_z$ in Fig. S12 where linear fits to $\Delta E_Z = g_v \mu_B B_z$ have been used to extract the $g_v$ for the $G = 8$, 12, and $16 \times e^2/h$ subbands.

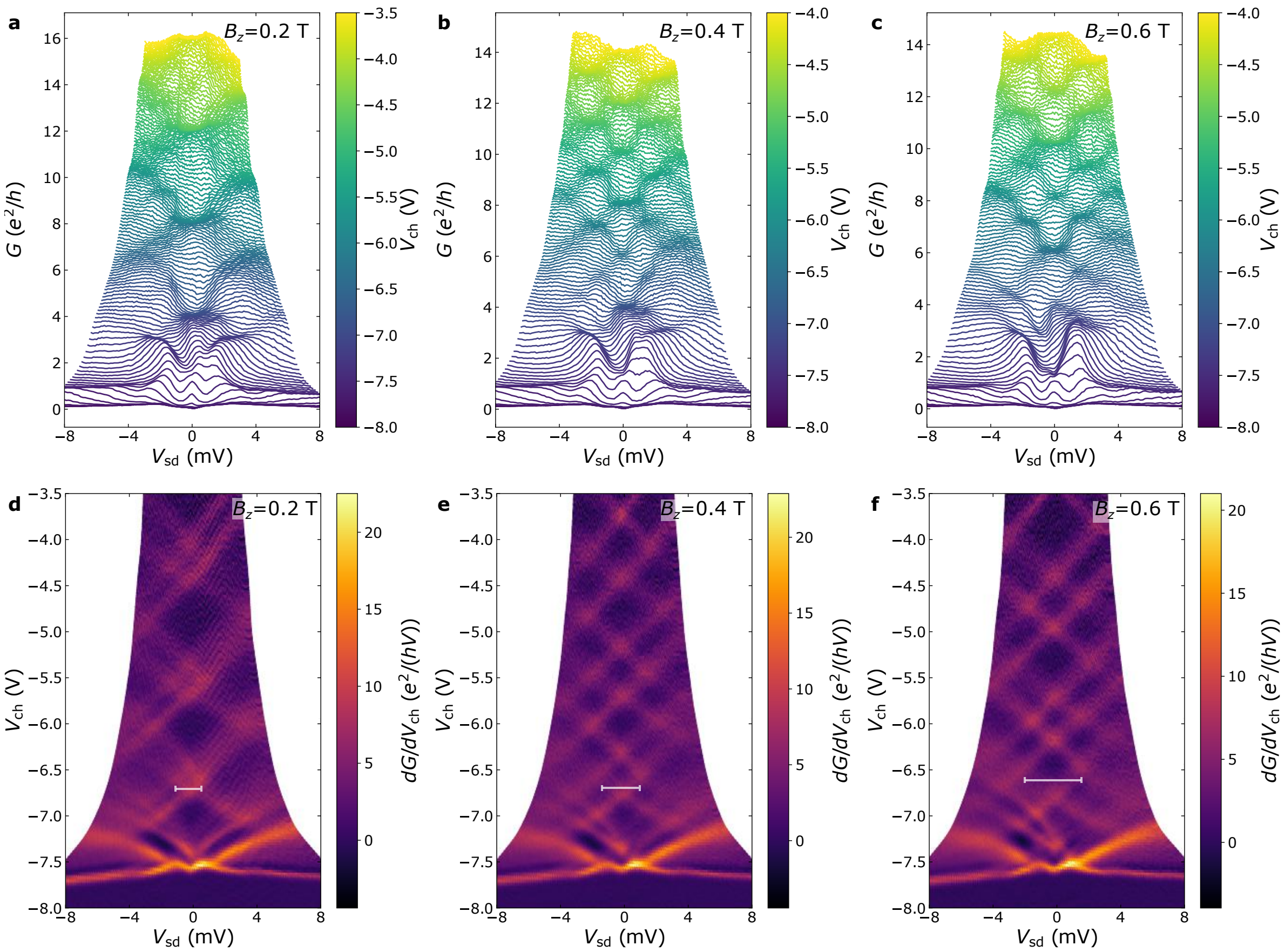


Figure S11. Effect of an out-of-plane magnetic field on the $V_{\rm sd}$ and $V_{\rm ch}$ dependence of the QPC conductance. (a-c) QPC conductance vs. $V_{\rm sd}$ at different $V_{\rm ch}$ values for $B_z = 0.2$, 0.4, and 0.6 T, respectively. (d-f) QPC transconductance vs. $V_{\rm sd}$ and $V_{\rm ch}$ for $B_z = 0.2$, 0.4, and 0.6 T, respectively. The white horizontal lines indicate the $B_z$-induced second subband splitting.

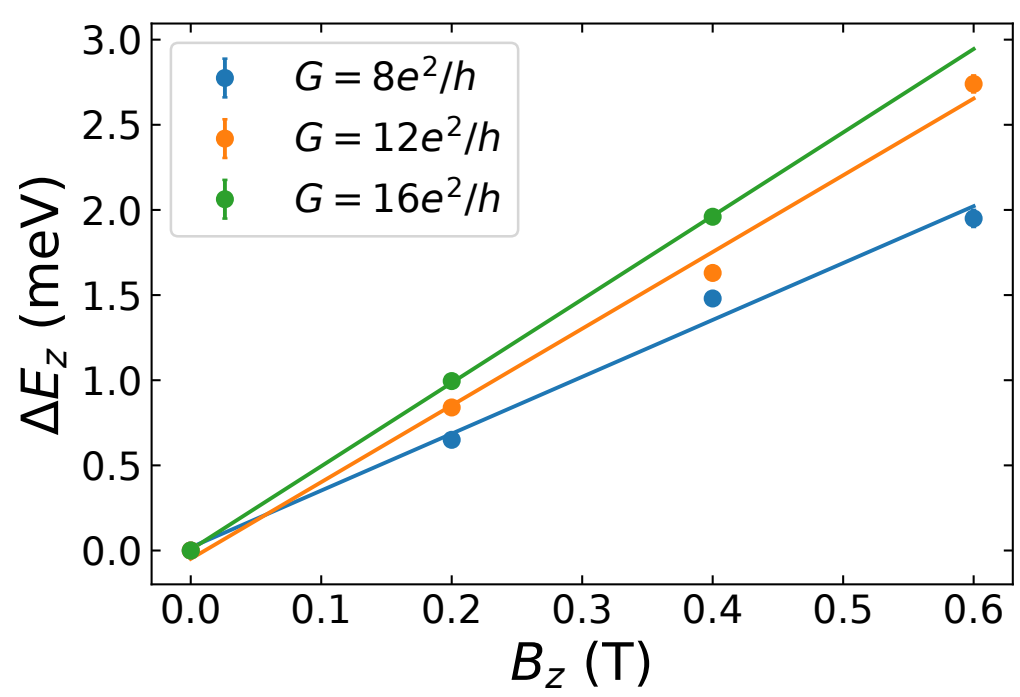


Figure S12. $B_z$-induced subband splitting ($\Delta E_z$) obtained from Fig. S11 for the first three subbands (scatter). The lines are fits to $\Delta E_Z = g_v \mu_B B_z$, resulting in $g_v = 58 \pm 4$, $78 \pm 4$, and $85 \pm 1$ for the $G = 8$, 12, and $16 \times e^2/h$ subbands, respectively.

[1] S. Cronenwett, H. Lynch, D. Goldhaber-Gordon, L. Kouwenhoven, C. Marcus, K. Hirose, N. Wingreen, and V. Umansky, Low-temperature fate of the 0.7 structure in a point contact: A kondo-like correlated state in an open system, Physical Review Letters **88**, 226805 (2002).

[2] H. Overweg, H. Eggimann, X. Chen, S. Slizovskiy, M. Eich, R. Pisoni, Y. Lee, P. Rickhaus, K. Watanabe, T. Taniguchi, *et al.*, Electrostatically induced quantum point contacts in bilayer graphene, Nano Letters **18**, 553 (2018).

[3] D. Goldhaber-Gordon, J. Göres, M. Kastner, H. Shtrikman, D. Mahalu, and U. Meirav, From the kondo regime to the mixed-valence regime in a single-electron transistor, Physical Review Letters **81**, 5225 (1998).

[4] M. Gruber, A. Weismann, and R. Berndt, The kondo resonance line shape in scanning tunnelling spectroscopy: instrumental aspects, Journal of Physics: Condensed Matter **30**, 424001 (2018).

[5] B. J. van Wees, L. Kouwenhoven, E. Willems, C. Harmans, J. Mooij, H. Van Houten, C. Beenakker, J. Williamson, and C. Foxon, Quantum ballistic and adiabatic electron transport studied with quantum point contacts, Physical Review B **43**, 12431 (1991).

[6] J. Göres, D. Goldhaber-Gordon, S. Heemeyer, M. Kastner, H. Shtrikman, D. Mahalu, and U. Meirav, Fano resonances in electronic transport through a single-electron transistor, Physical Review B **62**, 2188 (2000).

[7] M. Iqbal, R. Levy, E. Koop, J. Dekker, J. De Jong, J. Van Der Velde, D. Reuter, A. Wieck, R. Aguado, Y. Meir, *et al.*, Odd and even kondo effects from emergent localization in quantum point contacts, Nature **501**, 79 (2013).

[8] H. Overweg, A. Knothe, T. Fabian, L. Linhart, P. Rickhaus, L. Wernli, K. Watanabe, T. Taniguchi, D. Sánchez, J. Burgdörfer, *et al.*, Topologically nontrivial valley states in bilayer graphene quantum point contacts, Physical Review Letters **121**, 257702 (2018).

[9] R. Kraft, J. Mohrmann, R. Du, P. B. Selvasundaram, M. Irfan, U. N. Kanilmaz, F. Wu, D. Beckmann, H. Von Löhneysen, R. Krupke, *et al.*, Tailoring supercurrent confinement in graphene bilayer weak links, Nature Communications **9**, 1722 (2018).

[10] A. Knothe and V. Fal'ko, Influence of minivalleys and berry curvature on electrostatically induced quantum wires in gapped bilayer graphene, Physical Review B **98**, 155435 (2018).

[11] Y. Lee, A. Knothe, H. Overweg, M. Eich, C. Gold, A. Kurzmann, V. Klasovika, T. Taniguchi, K. Wantanabe, V. Fal'ko, *et al.*, Tunable valley splitting due to topological orbital magnetic moment in bilayer graphene quantum point contacts, Physical Review Letters **124**, 126802 (2020).
